# Going Up Inflame: Reviewing the Underexplored Role of Inflammatory Programming in Stress-Induced Intrauterine Growth Restricted Livestock

**DOI:** 10.3389/fanim.2021.761421

**Published:** 2021-11-04

**Authors:** Zena M. Hicks, Dustin T. Yates

**Affiliations:** Stress Physiology Laboratory, Department of Animal Science, University of Nebraska-Lincoln, Lincoln, NE, United States

**Keywords:** adaptive fetal programming, developmental origins of health and disease, DOHAD, fetal growth restriction, intrauterine growth restriction, IUGR, low birthweight, metabolic programming

## Abstract

The impact of intrauterine growth restriction (IUGR) on health in humans is well-recognized. It is the second leading cause of perinatal mortality worldwide, and it is associated with deficits in metabolism and muscle growth that increase lifelong risk for hypertension, obesity, hyperlipidemia, and type 2 diabetes. Comparatively, the barrier that IUGR imposes on livestock production is less recognized by the industry. Meat animals born with low birthweight due to IUGR are beset with greater early death loss, inefficient growth, and reduced carcass merit. These animals exhibit poor feed-to-gain ratios, less lean mass, and greater fat deposition, which increase production costs and decrease value. Ultimately, this reduces the amount of meat produced by each animal and threatens the economic sustainability of livestock industries. Intrauterine growth restriction is most commonly the result of fetal programming responses to placental insufficiency, but the exact mechanisms by which this occurs are not well-understood. In uncompromised pregnancies, inflammatory cytokines are produced at modest rates by placental and fetal tissues and play an important role in fetal development. However, unfavorable intrauterine conditions can cause cytokine activity to be excessive during critical windows of fetal development. Our recent evidence indicates that this impacts developmental programming of muscle growth and metabolism and contributes to the IUGR phenotype. In this review, we outline the role of inflammatory cytokine activity in the development of normal and IUGR phenotypes. We also highlight the contributions of sheep and other animal models in identifying mechanisms for IUGR pathologies.

## INTRODUCTION

Intrauterine growth restriction (IUGR) frequently results from stress-induced placental insufficiency, which reduces O_2_ and nutrients available to the fetus and consequently stunts growth of the highly metabolic fetal muscle tissues (Yates et al., 2018; Pendleton et al., 2021). In livestock, low birthweight due to stress-induced IUGR causes substantial economic losses for the industry due to greater neonatal mortality, less metabolic efficiency, and lower carcass quality (Reynolds et al., 2010; Liu and He, 2014; Ji et al., 2017; Yates et al., 2018). Estimates put low birthweight-related losses at approximately 8% of the potential annual product for US producers and up to 20% of the global annual product (Wu et al., 2006; Flinn et al., 2020). Thrifty metabolic adaptations to muscle, adipose, pancreatic islets, and other tissues cause IUGR-born offspring to be disadvantaged at birth due to insufficient energy stores and poor thermoregulation, which often results in reduced nursing success and a failure to thrive throughout the early neonatal period (Dwyer et al., 2016; Yates et al., 2018). Intrauterine growth restriction-born offspring that survive exhibit reduced feed efficiency, making it cost more to reach proper harvest weight (Bérard et al., 2008; Yates et al., 2018; Gibbs et al., 2020). Thrifty programming also manifests as reduced muscle growth capacity and increased fat deposition beginning at the juvenile age (Greenwood et al., 2000; Yates et al., 2012; Gibbs et al., 2020), which lowers carcass yield and affects meat quality parameters such as tenderness, muscle pH, meat color, and cooking loss (Liu and He, 2014; Matyba et al., 2021). Intrauterine growth restriction also afflicts human pregnancies (Saleem et al., 2011; Nardozza et al., 2017), and global estimates indicate that upward of 53 million infants are born IUGR each year (Sedgh et al., 2014). These babies are at increased risk for perinatal morbidity and mortality (Aucott et al., 2004; Alisi et al., 2011; Alisjahbana et al., 2019) as well as for lifelong health problems such as asthma, type 2 diabetes, cardiovascular disease, obesity, and neurocognitive disorders that begin in early childhood and reduce life expectancy and quality (Nardozza et al., 2017; Darendeliler, 2019; Xing et al., 2020; Briana and Malamitsi-Puchner, 2021).

By the late 1950s, the scientific community had recognized that individuals with metabolic diseases often exhibited physiological indicators of metabolic dysfunction by the time they were neonates (Neel, 1962). However, it was the work of Hales and Barker in the early 1990s and the subsequent publication of their Thrifty Phenotype hypothesis that popularized the idea of a link between fetal developmental programming and lifelong metabolic health (Hales et al., 1991; Hales and Barker, 1992). In the almost three decades since, a number of studies in humans and animal models have advanced this theory with details of how stress before birth causes tissue-specific adaptive programming of growth and metabolism (Morrison, 2008; Posont et al., 2017; Yates et al., 2018; Posont and Yates, 2019; Pendleton et al., 2021). These nutrient-sparing fetal adaptations help to increase the chances for survival *in utero* but also create permanent metabolic changes that are detrimental to long-term health of the offspring (Sharma et al., 2016a; Kesavan and Devaskar, 2019; Posont and Yates, 2019). Identifying the exact mechanistic facilitators of these changes has been challenging, but one likely potential mechanism that has recently come to light is inflammatory programming (Posont et al., 2018, 2021; Cadaret et al., 2019). This review highlights findings that provide insight for how fetal stress leads to programmed changes in inflammatory pathways that regulate growth and metabolism, with a primary focus on the implications for meat animals.

## CAUSES AND PROGRESSION OF IUGR

### IUGR Is the Developmental Response to Maternofetal Stress

Clinically, IUGR [alternatively, fetal growth restriction (Nardozza et al., 2017)] is characterized by less growth of the fetus or fetal tissues relative to expected growth potential (Sharma et al., 2016b; Reynolds et al., 2019). It is a pathological condition brought on by fetal nutrient restriction or other stress, although genetic abnormalities can increase the risk (Sharma et al., 2017). In the field of developmental origins of health and disease (DOHaD), IUGR is often used to describe the broader pathological phenotype resulting from chronic fetal stress, which typically (but not always) includes measurable reductions in placental function and birthweight (Sharma et al., 2016b). A number of different maternal conditions can result in placental stunting when they coincide with critical windows for placental growth and development (Sharma et al., 2016b; Yates et al., 2018). In livestock, common factors include environmental stress, illness or forage toxicity, nutritional imbalances, young age of the dam, uterine trauma from previous pregnancies, twin/triplet pregnancies, and side effects from artificial insemination or embryo transfer (Greenwood and Bell, 2003; Greenwood and Cafe, 2007). Such stressors redirect maternal blood flow from the gravid uterus, thus reducing nutritional support for placental hyperplasia and vasculogenesis (Burton and Jauniaux, 2018). Placental functional capacity is determined in large part by the successful establishment of uteroplacental circulation via the rapid development of villous blood vessels beginning around the end of the first trimester and continuing throughout most of the second trimester (Regnault et al., 2003; Burton and Jauniaux, 2018). Indeed, it is during this critical window that the placenta is most vulnerable to insults that may lead to reduction in its vasculature, surface area, and transport proteins needed for maternal-fetal nutrient exchange (Regnault et al., 2005; Burton and Jauniaux, 2018). During such insults, placental tissues are typically characterized by unusually high levels of inflammation, oxidative stress, and apoptotic cells (Cotechini and Graham, 2015; Burton and Jauniaux, 2018).

The diminished maternofetal interface associated with placental insufficiency ultimately reduces O_2_ transfer to the fetus, and reductions in placental glucose and amino acid transporters likewise reduce fetal availability of these nutrients (Brown, 2014; Yates et al., 2018; Beede et al., 2019). Indeed, fetal hypoxemia and hypoglycemia in heat stress-induced sheep models of placental insufficiency can exceed 50% reductions near term (Macko et al., 2016; Wai et al., 2018; Stremming et al., 2020), creating a clear need for changes in metabolic processes and growth trajectories (Lackman et al., 2001; Gagnon, 2003). The phenomenon of fetal hypoglycemia can be partially mimicked by sustained maternal undernutrition, which can decrease fetal growth despite little or no impact on the size or vascularity of the placenta (Lemley et al., 2012; Eifert et al., 2015; Edwards et al., 2020; Contreras-Correa et al., 2021). Interestingly, diminished placental transfer of amino acids due to downsizing of system A and L transporters does not necessarily manifest in reduced fetal blood concentrations (Pantham et al., 2016; Wai et al., 2018), as the IUGR fetus compensates by slowing its protein utilization and accretion rates (Rozance et al., 2018; Wai et al., 2018; Stremming et al., 2020).

The hypoxemic and hypoglycemic conditions resulting from placental insufficiency cause a robust hormone-driven stress response by the fetus. Low blood O_2_ concentration detected by O_2_-sensitive K^+^ channels on the chromaffin cells of the adrenal medulla stimulates secretion of the catecholamines, norepinephrine, and epinephrine (Adams and McMillen, 2000), inducing the hallmark hypercatecholaminemia that progressively worsens over the third trimester of pregnancy. Catecholamines act as strong inhibitors of insulin secretion, which together with hypoglycemia results in a chronic state of fetal hypoinsulinemia (Chen et al., 2017; Limesand and Rozance, 2017). Fetal hypoxemia also leads to an increase in circulating inflammatory cytokines (Krajewski et al., 2014; Visentin et al., 2014), which will be discussed in detail in later sections. Additional inflammatory components such as chemokine C-C motif ligand 16 (CCL16) and acute phase protein C-reactive protein (CRP) have also been found to be increased in IUGR fetuses (Makikallio et al., 2012; Visentin et al., 2014).

### IUGR Impairs Growth Capacity and Metabolic Function

In most cases, the fetus can survive unfavorable conditions created by placental insufficiency by altering the development of several growth and metabolic processes in a way that reduces nutrient demands (Gagnon, 2003). First, the combined endocrine response to low fetal blood O_2_ content causes a redirection of blood flow away from skeletal muscle and other less vital tissues to maintain support for the brain, liver, adrenals, and pancreas (Gagnon, 2003; Poudel et al., 2015). Indeed, greater vascular resistance can reduce blood flow to muscle-dense areas such as the hindlimb by as much as 45%, which in turn reduces O_2_ delivery by up to 40% (Rozance et al., 2018). Secondly, hypoinsulinemia reduces glucose utilization by insulin-sensitive muscle tissues (Davis et al., 2020). Interestingly, this can lead to transient enhancement of insulin sensitivity in the early neonatal period as a compensatory response (Soto et al., 2003; Ong et al., 2004). However, this wanes relatively quickly (Mericq et al., 2005), exposing underlying impairments in insulin action (Jensen et al., 2002).

#### Poor Skeletal Muscle Growth Leads to Asymmetric Body Composition

**FIGURE 1 |:**
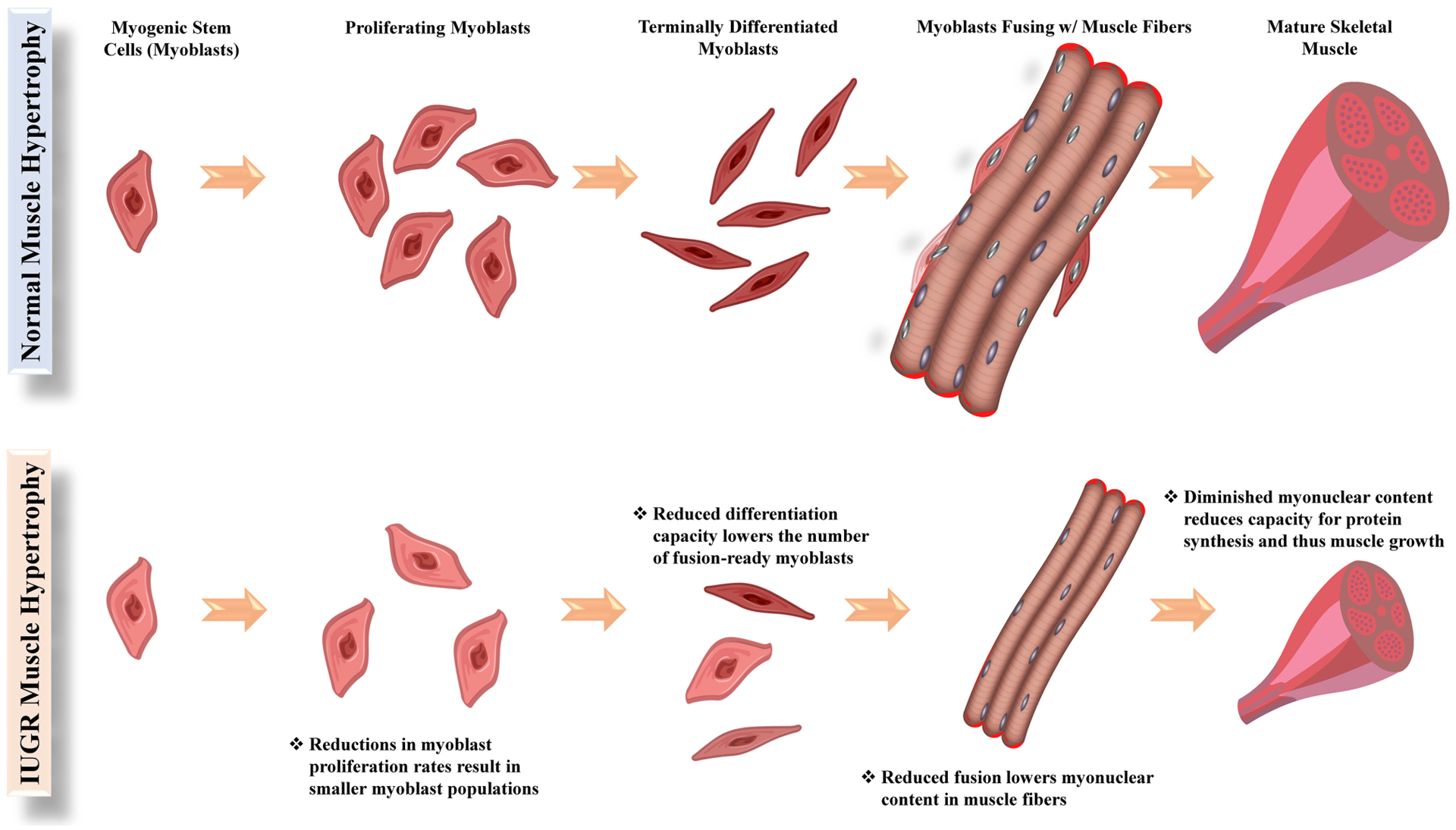
Functional steps of myoblasts (muscle stem cells) and their facilitation of hypertrophic growth in normal and IUGR skeletal muscle.

The reappropriation of nutrients away from skeletal muscle in the IUGR fetus causes the development more conservative muscle growth rates that are apparent in late gestation but also persist throughout the lifetime of the animal. Indeed, IUGR fetal sheep and rats were found to have smaller cross-sectional areas for all muscle fiber types (Yates et al., 2016; Cadaret et al., 2019a), indicating that less muscle hypertrophy was occurring during gestation. Intrinsic functional deficits in muscle stem cells called myoblasts are a major underlying factor for impaired muscle growth capacity (Yates et al., 2014; Soto et al., 2017; Posont et al., 2018). In ruminants and humans, muscle hyperplasia is completed early in the third trimester, and subsequent muscle growth is the result of myofiber hypertrophy (Maier et al., 1992; Wilson et al., 1992). Indeed, postnatal muscle growth results from the accumulation of new nuclei within muscle fibers via fusion of myoblasts, which increases capacity for fiber protein synthesis (Allen et al., 1979; Davis and Fiorotto, 2009). Some fetal myoblasts form quiescent populations between the sarcolemma and the basal lamina of muscle fibers. These latent myoblast populations are called satellite cells and can later be activated to facilitate further muscle growth (Davis and Fiorotto, 2009; Yin et al., 2013). Before fusing, myoblasts undergo several cycles of proliferation followed by terminal differentiation, both of which are rate-limiting functional steps for muscle growth (Allen et al., 1979; Allen and Boxhorn, 1989). However, myoblasts from IUGR fetal sheep and rats were found to exhibit reduced proliferation and differentiation capabilities (Yates et al., 2014; Soto et al., 2017; Posont et al., 2018; Cadaret et al., 2019a), leading to reduced muscle mass at birth and throughout postnatal life, as illustrated in Figure 1.

Offspring born with low birthweight due to IUGR initially continue to exhibit slower postnatal growth. For example, lambs born IUGR due to maternal heat stress or maternofetal inflammation remained about 20% smaller at 30 days of age, with comparable reductions in average daily gain (Cadaret et al., 2019b; Yates et al., 2019; Posont et al., 2021). As IUGR-born offspring reach the juvenile stage, many begin to exhibit postnatal catch-up growth, whereby their bodyweights equalize with uncompromised herdmates. Indeed, bodyweights and average daily gain for lambs born IUGR due to maternal heat stress were reduced by only about 12% by 60 days of age (Gibbs et al., 2020), and IUGR-born beef cattle were about 8% lighter at 30 months of age (Greenwood et al., 2005; Greenwood and Cafe, 2007). However, this does not equate to recovery of muscle growth, and thus body composition remains impaired; 60-day old IUGR lambs had smaller loin eye areas, reduced muscle protein, and greater fat-to-protein ratios (Gibbs et al., 2020), and 30-month old IUGR beef cattle had smaller carcass weight, ribeye area, and *longissimus* muscle weight, resulting in less retail yield (Greenwood and Cafe, 2007). Estimates from these cattle indicate that each 1-kg reduction in birthweight equated to a 4.4-kg reduction in slaughter weight (Robinson et al., 2013; Greenwood and Bell, 2019).

#### Nutrient-Sparing Adaptations Reduce Muscle Glucose Metabolism

In concert with more conservative muscle growth, the IUGR fetus undergoes a glucose-sparing shift in muscle metabolism characterized by reduced oxidation and greater glycolytic lactate production. When IUGR fetal sheep were made hyperglycemic or hyperinsulinemic near term, whole-body glucose oxidation was decreased even though whole-body glucose utilization remained unchanged (Limesand et al., 2007; Brown et al., 2015). Subsequent sheep studies confirmed that the reduction in glucose oxidation rates were muscle-specific and persisted after birth (Cadaret et al., 2019b; Yates et al., 2019; Gibbs et al., 2021; Posont et al., 2021). Four-fold greater circulating lactate concentrations together with greater hepatic expression of gluconeogenic genes in IUGR fetal sheep (Brown et al., 2015) indicate that lactate produced in greater amounts by IUGR skeletal muscle supports hepatic glucose production. This process, called the Cori cycle, benefits the nutrient-restricted IUGR fetus by engaging an otherwise inactive source for glucose (Thorn et al., 2009; Davis et al., 2021). However, it is important to note that the reduction in glucose oxidation arises from a programmed change in mitochondrial functional capacity that does not appear to be reversible. Although pyruvate dehydrogenase functional activity was increased in IUGR fetal sheep muscle (Pendleton et al., 2019), mitochondrial O_2_ consumption and electron transport chain Complex I activity were impaired (Pendleton et al., 2020). Gene expression for isocitrate dehydrogenase, mitochondrial pyruvate carrier, and other integral components of mitochondrial oxidative metabolism were also reduced in IUGR muscle, whereas gene expression for lactate dehydrogenase B (converts pyruvate to lactate) was increased 2.5-fold (Pendleton et al., 2020). It is worth noting that reduced glucose oxidation rates do not appear to be offset by compensatory amino acid oxidation (Pendleton et al., 2021). In fact, oxidation rates for the representative amino acid, leucine, were slightly reduced in IUGR fetal sheep (Brown et al., 2012; Wai et al., 2018). Moreover, impaired glucose oxidation coincided with reduced proportions of oxidative myofibers in hindlimb muscles of IUGR fetal sheep (Yates et al., 2016).

#### Insulin Signaling Is Impaired in IUGR Skeletal Muscle

Growth and metabolic deficits in IUGR skeletal muscle are at least partially a product of disruptions in insulin signaling through Akt-mediated pathways. Insulin is a primary promotor of muscle growth, as it enhances protein synthesis (Davis and Fiorotto, 2009) and is a well-established stimulator of proliferation and differentiation in adult myoblasts (Allen et al., 1985; Sumitani et al., 2002). More recent studies found that hyperinsulinemia also increases myoblast function in fetal sheep (Brown et al., 2016b; Soto et al., 2017). Additionally, skeletal muscle is the primary tissue for insulin-mediated glucose uptake from the blood (Baron et al., 1988; Brown, 2014). This is facilitated when circulating insulin binds to receptors on the muscle fiber surface and initiates rapid mobilization of sequestered glucose transporter 4 (Glut4) to the cell membrane, where it facilitates glucose diffusion into the cell (Kubota et al., 2011). In addition to its effects on glucose uptake, insulin stimulation increased skeletal muscle glucose oxidation rates 1.5- to 4-fold in fetal sheep (Brown et al., 2015; Cadaret et al., 2019) and 2- to 8-fold in growing lambs (Barnes et al., 2019; Cadaret et al., 2019b; Swanson et al., 2020; Posont et al., 2021). However, several studies have indicated that insulin/Akt signaling is impaired in IUGR muscle. Insulin activates Akt by serine^463^ phosphorylation, but the proportion of phosphorylated Akt was reduced in *flexor digitorum superficialis* muscle from IUGR fetal and neonatal sheep (Cadaret et al., 2019; Posont et al., 2021). This deficit was observed at both low and high insulin concentrations and did not coincide with any reduction in insulin receptor content (Thorn et al., 2009; Yates et al., 2019). Intrauterine growth restriction skeletal muscle also exhibited reduced content of the insulin-sensitive glucose transporter, Glut4, before and after birth (Limesand et al., 2007; Yates et al., 2019), likely due to epigenetic mechanisms such as DNA methylation at the Glut4 promoter region or histone modifications (Raychaudhuri et al., 2008; Wang et al., 2016). Like insulin, the influence of IGF-1 is also diminished in the IUGR fetus, as circulating IGF-1 concentrations and skeletal muscle signaling components are reduced (Thorn et al., 2009; Rozance et al., 2018).

#### Pancreatic Islet Dysfunction Contributes to Metabolic Deficits

Stress conditions resulting from placental insufficiency induce programming in other tissues that further compounds muscle-centric dysfunction. Chief among these affected tissues are pancreatic islets, which are diminished in both development and functionality (Boehmer et al., 2017). Near term, IUGR fetal sheep islets are reduced in size by 40% (Rozance et al., 2015; Brown et al., 2016a), and β cell mass is reduced by 60% due to a 40–60% reduction in mitosis (Limesand et al., 2005; Brown et al., 2016a). In addition to being smaller, these β cell populations are less productive, as IUGR fetal islets contain only about 20% the amount of insulin found in normal fetal islets (Limesand et al., 2006). Deficits in islet microanatomy are preceded by insufficient islet vascular formation, which is observable shortly after the start of the third trimester (Rozance et al., 2015). Islet under-development may be due in part to less profound HGF paracrine activity originating from islet endothelial cells (Rozance et al., 2015; Brown et al., 2016a), which appears necessary for β cell development and performance (Dai et al., 2005; Johansson et al., 2009). Like muscle, IUGR fetal islets are also less capable of glucose oxidation, which is the impetus for glucose-stimulated insulin secretion (Limesand et al., 2006). These programmed deficits persist in offspring, as islets from IUGR-born lambs maintained substantially reduced insulin content and glucose stimulus-secretion coupling (Yates et al., 2019), leading to impairments in glucose-stimulated insulin secretion that are comparable to the fetus (Cadaret et al., 2019b; Yates et al., 2019). Interestingly, α cell mass is reduced in IUGR fetal islets, but not to the same magnitude observed for β cells. Moreover, their capacity to appropriately secrete glucagon appears to be unaffected (Limesand et al., 2005).

## THE ROLE OF INFLAMMATORY CYTOKINES IN IUGR OUTCOMES

### Cytokines Regulate Muscle Growth and Metabolism

Cytokines are a broad class of peptide chemical messengers produced by a wide array of cell and tissue types, often in response to the presence of pathogens, toxins, free radicals, and stress (Tracey and Cerami, 1993; Reid and Li, 2001). Among their other immune functions, inflammatory cytokines modify metabolic activity in muscle and other tissues in order to reappropriate O_2_, glucose, and protein (Li and Reid, 2001; Cadaret et al., 2017). They are also potent regulators of muscle growth via their complex impact on myoblast function and insulin sensitivity (Otis et al., 2014; Posont et al., 2018). This makes the broad cytokine milieu integral to metabolic homeostasis and, in turn, general metabolic health, as summarized in Table 1.

Tumor necrosis factor α (TNFα) is perhaps the most comprehensively studied inflammatory cytokine. It is produced in greatest quantities by circulating monocytes and their intra-tissue counterparts, macrophages, but is also produced by glycolytic skeletal muscle fibers and fat cells (Tracey and Cerami, 1993; Li and Reid, 2001; Plomgaard et al., 2005; Dyck et al., 2006). Basal circulating TNFα concentrations are typically low but increase rapidly and profoundly when stimulated (Tracey and Cerami, 1993; Li and Reid, 2001). Pathological metabolic states such as excessive fat deposition, insulin resistance, and hyperglycemia are associated with substantially greater production and secretion of TNFα (Saghizadeh et al., 1996; Lo et al., 2007), as is pathological muscle atrophy (Li and Reid, 2001). Most cell types express one or both of two surface TNFα receptor isoforms (TNFR1, TNFR2), and TNFR1 is predominant for muscle (Popa et al., 2007). Once activated by TNFα, the intracellular domain of TNFR1 binds and activates the downstream TNFR1-associated death domain (TRADD) proteins (Popa et al., 2007), which in turn activate Fas-associated protein with death domain (FADD) pathways and TRAF2/NFκB pathways (Popa et al., 2007). In skeletal muscle, these pathways are most associated with protein catabolism, lipolysis, and metabolic suppression (Cheema et al., 2000; Li and Reid, 2001; Popa et al., 2007). They also decrease synthesis of the myofibril components myosin and actin as well as sarcoplasmic proteins (Cheema et al., 2000; Li and Reid, 2001; Lang et al., 2002). In differentiating myoblasts, TNFα inhibits MyoD expression, which impedes their progression, and in mature fibers it increases protein catabolism, which reduces the content of myosin and other integral proteins for muscle function (Li and Reid, 2001). Both of these outcomes appear to be mediated by canonical NFκB pathways (Li and Reid, 2001; Remels et al., 2015). In mature skeletal muscle, TNFα-activated NFκB pathways increase the proportion of glucose undergoing glycolytic lactate production by increasing activity of HIF-1α, which is concurrent with reduced glycogen synthesis (Boscá and Corredor, 1984; Rhoades et al., 2005; Remels et al., 2015). The effects of TNFα on glucose oxidation rates are more complex, as the cytokine appears to be stimulatory during acute exposure but inhibitory when exposure is sustained (Gao et al., 2012; Cadaret et al., 2017, 2019). In addition to direct effects on muscle growth and metabolism, TNFα also impairs insulin sensitivity. In rats, TNFα diminished the effects of insulin on skeletal muscle glucose uptake by 50% (Youd et al., 2000; Li and Reid, 2001), perhaps by increasing the content of diacylglyceride, a potent activator of the insulin antagonist protein kinase C (Bruce and Dyck, 2004; Dyck et al., 2006). In pancreatic islet cells, TNFα exposure was shown to reduce glucose metabolism in β cells and, in turn, glucose-stimulated insulin secretion (Oleson et al., 2015).

The interleukin IL-6 is produced by leukocytes and muscle cells, often in response to rising TNFα concentrations (Tracey and Cerami, 1993; Haddad et al., 2005). Consequently, circulating IL-6 concentrations follow similar patterns as TNFα during sub-acute and chronic inflammatory conditions (Wolsk et al., 2010). When bound, the soluble IL-6 receptor (IL6R) forms a heterodimer with the downstream messenger gp130 (Wang et al., 2013), which primarily activates the JAK/STAT3 pathway but can also activate PI3K/Akt/mTOR and Ras/Raf/MEK/ERK pathways (Wang et al., 2013; Johnson et al., 2018). Elevated IL-6 activity limits hypertrophic muscle growth by interfering with growth hormone and IGF-I activity and also increases muscle protein catabolism, thus contributing to muscle atrophy (Haddad et al., 2005; Bodell et al., 2009). Although it may increase myoblast proliferation at some concentrations, IL-6 also reduces the progression of differentiation in primary fetal sheep myoblasts (Haddad et al., 2005; Posont et al., 2018). The predominant aspects of metabolic regulation by IL-6 are similar to those of TNFα. First, the nature of its effects on skeletal muscle appear dependent on the magnitude and duration of exposure, as sustained exposure is substantially more detrimental. Additionally, IL-6 is associated with pathological metabolic states, presumably due to its propensity to decrease skeletal muscle carbohydrate metabolism in favor of fatty acid oxidation and to disrupt insulin signaling (Bruce and Dyck, 2004; Wolsk et al., 2010; Knudsen et al., 2017). Finally, IL-6 is detrimental to pancreatic islet function, as overexpression of IL-6 in β cells resulted in alterations to islet structure, increased fibrosis, and decreased insulin production (Campbell et al., 1994).

Additional inflammatory cytokines appear to have roles in muscle regulation but have been less extensively studied. For example, IL-1β is involved in collagen degradation, muscle protein catabolism, and branched-chain amino acid metabolism (Nawabi et al., 1990; Dinarello, 2000; Li et al., 2009). Consequently, it is associated with reduced myofiber width, myofibril construction, and actin content (Li et al., 2009). In islets, IL-1β promotes β cell apoptosis, which impairs glucose-stimulated insulin secretion (Harms et al., 2015; Oleson et al., 2015). IL-18, IL-8, and TWEAK appear to have similar roles in regulating muscle growth and metabolism.

### Inflammatory Tone Is Increased in the IUGR Fetus

Studies in a multitude of mammalian species show that IUGR fetuses exhibit greater circulating leukocyte and cytokine concentrations, which correlate closely with hypoxemia (Romero et al., 2007; Guo et al., 2010; Cadaret et al., 2019; Oh et al., 2019). Increased TNFα, IL-6, and IL-18 were observed in cord blood of IUGR infants at delivery and in blood serum at 24 h after delivery (Krajewski et al., 2014; Visentin et al., 2014). In fact, high concentrations of inflammatory cytokines in cord blood are considered reliable clinical markers for diagnosing fetal inflammatory response syndrome (FIRS) (Kemp, 2014). In IUGR fetal sheep, greater circulating TNFα in the mid-third trimester coincided with increased monocytes, granulocytes, and total white blood cells (Cadaret et al., 2019). Similarly, IUGR fetal rodents exhibited elevated blood concentrations of TNFα, IL-6, and IFNγ, as well as greater leukocyte activity (Hudalla et al., 2018; Cadaret et al., 2019a). In addition to circulating concentrations, cytokine expression is elevated in IUGR tissues including lungs, brain, skeletal muscle, and white blood cells (Kemp, 2014; Cadaret et al., 2019a), but not necessarily in pancreatic islets (Kelly et al., 2017).

### IUGR Tissues Develop Enhanced Inflammatory Sensitivity

**FIGURE 2 |:**
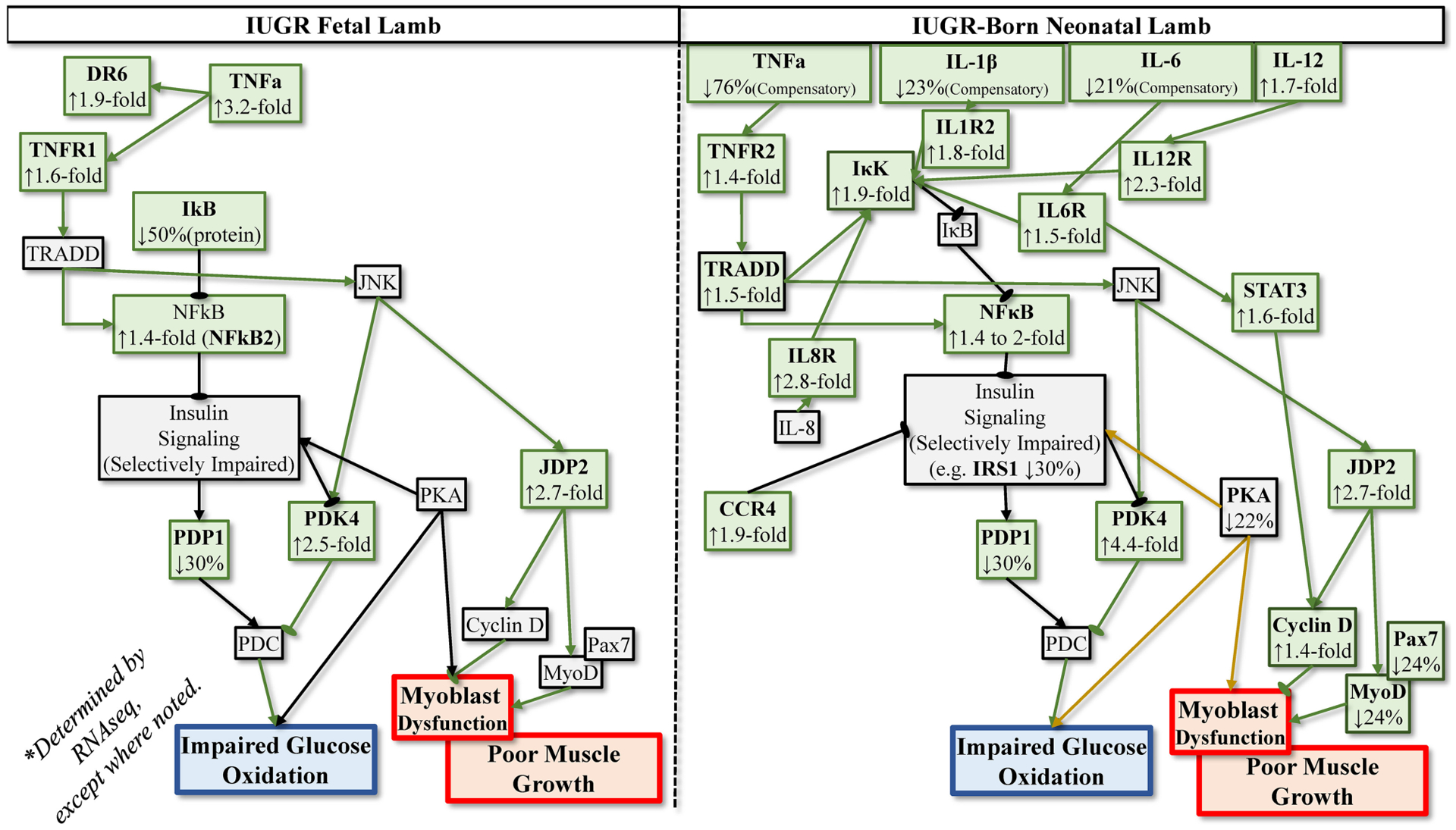
Postulated programming of enhanced sensitivity to inflammatory cytokines based on transcriptomics analyses of IUGR semitendinosus muscle from IUGR fetal sheep during late gestation and IUGR-born neonatal lambs at 1 month of age.

Some rodent models of IUGR indicate that greater circulating cytokine concentrations are maintained into adulthood. Indeed, IUGR-born rat and mice offspring exhibited greater circulating TNFα, IL-6, and IL-1β from birth to adulthood (Desai et al., 2009; Riddle et al., 2014; Chisaka et al., 2016; Oliveira et al., 2017). However, recent findings indicate that enhanced cytokine signaling pathways in muscle and other tissues maintain increased inflammatory tone even when elevated circulating cytokines subside after birth (Cadaret et al., 2019a; Posont et al., 2021). We have postulated that this enhanced responsiveness to cytokines contributes to the persistent dysregulation of muscle growth and metabolic function observed in IUGR-born offspring (Yates et al., 2018; Posont and Yates, 2019). At term, skeletal muscle from IUGR rat pups exhibited greater gene expression for TNFR1, IL6R, and Fn14 (TWEAK receptor) (Cadaret et al., 2019a). Moreover, muscle from IUGR-born mice and rats exhibited greater TNFα and IL-6 gene expression at 2 and 12 months after birth (Sutton et al., 2010; Tarry-Adkins et al., 2016). In sheep, proliferation and differentiation rates of primary IUGR fetal myoblast were reduced when exposed to basal or high TNFα or IL-6 concentrations (Posont et al., 2018). Additional data from these samples indicate increased gene expression for TNFR1 and IL6R in IUGR myoblasts and *semitendinosus* muscle, as well as reduced muscle IκBα protein content and increased c-Fos protein content (Posont, 2019). As neonates, IUGR-born lambs exhibited increased TNFR1 protein content in *semitendinosus* muscle and greater circulating concentrations of monocytes, granulocytes, and platelets (Posont et al., 2021). Transcriptomics were subsequently performed in muscle samples from these lambs, which indicate that gene expression for numerous components of TNFα, IL-6, IL-1β, and IL-12 pathways were upregulated (Yates et al., 2018; Cadaret, 2019), as summarized in Figure 2. This paralleled similar transcriptomics findings in skeletal muscle from IUGR fetal sheep (Cadaret et al., 2019; Posont and Yates, 2019). Interestingly, IUGR-born lambs also exhibited greater muscle IκBα protein content and a 50% reduction in circulating TNFα, perhaps as a compensatory mechanism for enhanced cytokine sensitivity (Posont et al., 2021). Although additional studies are needed to fully understand the magnitude and nature of inflammatory programming in IUGR skeletal muscle, it is clear that such enhanced activity would help to explain the deficits in myoblast function, muscle growth, body composition, insulin action, and metabolic efficiency described in earlier sections.

### Targeting Inflammatory Adaptations May Improve IUGR Outcomes

Inflammatory programming is likely one of several underlying mechanisms for IUGR-associated pathologies, but its ability to be targeted makes it of particular interest. In fact, several studies have provided fundamental evidence that treating IUGR fetuses and IUGR-born offspring with anti-inflammatory nutrients or pharmaceuticals can help to mitigate or improve growth and metabolic deficits. In mice, maternal supplementation of the anti-inflammatory nutraceutical folic acid reduced the frequency and severity of IUGR resulting from maternal inflammation (Zhao et al., 2013). The mice receiving folic acid also exhibited a less severe increase in amniotic concentrations of IL-6 and other cytokines. Although not assessed in muscle, the enhancement of cytokine signaling pathways observed in IUGR placental tissues was mitigated by folic acid (Zhao et al., 2013). In sheep, direct daily infusion of the anti-inflammatory nutraceutical eicosapentaenoic acid (EPA) into the bloodstream of IUGR fetuses during the mid-third trimester of gestation for 5 days resulted in less severe fetal hypoxemia, hypoglycemia, and hyperlactatemia (Beer et al., 2021). In addition, the greater lactate production observed for IUGR fetuses during hyperglycemia was improved substantially, perhaps indicating a less severe metabolic shift to glycolytic lactate production by muscle (Lacey et al., 2021). This coincided with an improvement in fetal growth and body symmetry. EPA infusion also improved whole hindlimb mass as well as loin and *flexor digitorum superficialis* muscle mass in the IUGR fetuses, which is indicative of improved muscle growth during late gestation (Lacey et al., 2021). It is worth noting that growth was not recovered for all muscles, which may have been due to the natural differences in insulin sensitivity and metabolic phenotypes among muscle groups (Kirchofer et al., 2002). After IUGR fetuses had been infused with EPA for 5 days, they exhibited improved basal and glucose-stimulated insulin secretion, indicating partial rescue of pancreatic islet function (Lacey et al., 2021). These fetuses also exhibited improvements in blood pH, HCO_3_, Na^+^, and Ca^++^ concentrations, which indicate improved fetal health and well-being (Lacey et al., 2021). In addition to nutrient compounds, maternal delivery of anti-inflammatory pharmaceuticals may also represent an effective intervention strategy. A recent clinical study showed that high doses of the non-steroidal anti-inflammatory drug (NSAID) aspirin taken by pregnant women during critical windows of fetal development reduced the frequency of IUGR (Roberge et al., 2017). Of course, the benefits of such drugs must be considered in combination with potential side effects for fetal development. Inflammatory programming can also be targeted in offspring, which is of particular interest in livestock. Dietary supplementation of the anti-inflammatory nutraceutical curcumin to IUGR-born neonatal pigs and mice mitigated the elevated concentrations of blood TNFα, IL-6, and IL-1β, which improved insulin sensitivity, lipid homeostasis, and neonatal growth (Niu et al., 2019a,b,c).

## Figures and Tables

**TABLE 1 |:** Summary of the major effects that key inflammatory cytokines elicit in tissues affecting growth and efficiency in meat animals.

Cytokine	Tissue		
	Skeletal muscle	Pancreatic islets	Other
TNFα	↓MyoD (Li and Reid, 2001; Alvarez et al., 2020) ↓Myoblast differentiation (Alvarez et al., 2020) ↓Myosin, actin, and sarcoplasmic proteins (Alvarez et al., 2020) ↑Protein catabolism (Cheema et al., 2000; Popa et al., 2007) ↑Glycolysis (Boscá and Corredor, 1984; Rhoades et al., 2005; Remels et al., 2015)	↓Glucose oxidation in β cells (Oleson et al., 2015) ↓Glucose-stimulated insulin secretion (Oleson et al., 2015) ↓Insulin sensitivity (Youd et al., 2000; Li and Reid, 2001)	↑Lipolysis in fat deposits (Cheema et al., 2000; Popa et al., 2007)
IL-6	↓Muscle hypertrophy (Haddad et al., 2005; Bodell et al., 2009) ↓Myoblast differentiation (Haddad et al., 2005; Posont et al., 2018) ↑Muscle atrophy (Haddad et al., 2005; Bodell et al., 2009)	Altered islet structure (Campbell et al., 1994) ↑Fibrosis (Campbell et al., 1994) Impaired insulin signaling (Bruce and Dyck, 2004; Wolsk et al., 2010; Knudsen et al., 2017)	↓GH and IGF-1 secretion and sensitivity, multiple tissues (Haddad et al., 2005; Bodell et al., 2009)
IL-1β	↑Protein catabolism (Nawabi et al., 1990; Dinarello, 2000; Li et al., 2009) ↓Myofiber width (Li et al., 2009) ↓Actin content (Li et al., 2009)	↑β cell apoptosis (Harms et al., 2015; Oleson et al., 2015) ↓Glucose-stimulated insulin secretion (Harms et al., 2015; Oleson et al., 2015)	Collagen degradation, multiple tissues (Nawabi et al., 1990; Dinarello, 2000; Li et al., 2009)

## IMPLICATIONS

The link between maternofetal stress, IUGR-induced low birthweight, and postnatal deficits in metabolic efficiency and growth potential are well-established in livestock, but scientific advancements regarding the molecular mechanisms underlying this link have been lacking. However, recent studies provide evidence that systemic fetal inflammation and developmental programming that enhances tissue sensitivity to inflammatory cytokines are contributing factors to growth and metabolic deficits, particularly those that are muscle centric. Although inflammatory programming is likely one of many underlying mechanisms, it is of particular interest to the livestock industry because it is both identifiable and treatable. Indeed, there are a number of currently marketed nutritional supplements and pharmaceuticals with anti-inflammatory properties, which could provide producers a number of options for mitigation and treatment strategies. Food animals born with low birthweight due to stress-induced IUGR are persistently one of the greatest barriers to US and global livestock production. Moreover, the emergence of climate change will likely increase the incidence of environmental stress on pregnant animals, creating a greater challenge for sustainable livestock production. As the global population continues to increase, recovering growth, efficiency, and meat production in low birthweight animals through practical management strategies represents one of the most realistic options for increasing meat production without greater land and water resource inputs. Although more work is warranted, early evidence indicates that treatment of enhanced inflammatory tone in IUGR-born animals may be an effective approach.

## References

[R1] Adams MB, and McMillen IC (2000). Actions of hypoxia on catecholamine synthetic enzyme mRNA expression before and after development of adrenal innervation in the sheep fetus. J. Physiol 529, 519–531. doi: 10.1111/j.1469-7793.2000.00519.x 11118487 PMC2270232

[R2] Alisi A, Panera N, Agostoni C, and Nobili V (2011). Intrauterine growth retardation and nonalcoholic fatty liver disease in children. Int. J. Endocrinol 2011, 269853. doi: 10.1155/2011/269853 22190925 PMC3235463

[R3] Alisjahbana B, Rivami DS, Octavia L, Susilawati N, Pangaribuan M, Alisjahbana A, (2019). Intrauterine growth retardation (IUGR) as determinant and environment as modulator of infant mortality and morbidity: the Tanjungsari Cohort Study in Indonesia. Asia Pac. J. Clin. Nutr 28(Suppl 1), S17–S31. doi: 10.6133/apjcn.201901_28(S1).0002 30729772

[R4] Allen RE, and Boxhorn LK (1989). Regulation of skeletal muscle satellite cell proliferation and differentiation by transforming growth factor-beta, insulin-like growth factor I, and fibroblast growth factor. J. Cell. Physiol 138, 311–315. doi: 10.1002/jcp.1041380213 2918032

[R5] Allen RE, Luiten LS, and Dodson MV (1985). Effect of insulin and linoleic acid on satellite cell differentiation. J. Anim. Sci 60, 1571–1579. doi: 10.2527/jas1985.6061571x 3894312

[R6] Allen RE, Merkel RA, and Young RB (1979). Cellular aspects of muscle growth - myogenic cell-proliferation. J. Anim. Sci 49, 115–127. doi: 10.2527/jas1979.491115x 500507

[R7] Alvarez AM, DeOcesano-Pereira C, Teixeira C, and Moreira V (2020). IL-1β and TNF-α modulation of proliferated and committed myoblasts: IL-6 and COX-2-derived prostaglandins as key actors in the mechanisms involved. Cells 9, 2005. doi: 10.3390/cells9092005 PMC7564831 32882817

[R8] Aucott SW, Donohue PK, and Northington FJ (2004). Increased morbidity in severe early intrauterine growth restriction. J. Perinatol 24, 435–440. doi: 10.1038/sj.jp.7211116 15116139

[R9] Barnes TL, Cadaret CN, Beede KA, Schmidt TB, Petersen JL, and Yates DT (2019). Hypertrophic muscle growth and metabolic efficiency were impaired by chronic heat stress, improved by zilpaterol supplementation, and not affected by ractopamine supplementation in feedlot lambs. J. Anim. Sci 97, 4101–4113. doi: 10.1093/jas/skz271 31410479 PMC6776279

[R10] Baron AD, Brechtel G, Wallace P, and Edelman SV (1988). Rates and tissue sites of non-insulin- and insulin-mediated glucose uptake in humans. Am. J. Physiol 255(6 Pt 1), E769–E774. doi: 10.1152/ajpendo.1988.255.6.E769 3059816

[R11] Beede KA, Limesand SW, Petersen JL, and Yates DT (2019). Real supermodels wear wool: summarizing the impact of the pregnant sheep as an animal model for adaptive fetal programming. Anim. Front 9, 34–43. doi: 10.1093/af/vfz018 31608163 PMC6777506

[R12] Beer HN, Lacey TA, Gibbs RL, Most MS, Hicks ZH, Grijalva PC, Petersen JL, and Yates DT (2021). Placental insufficiency improves in intrauterine growth-restricted fetal sheep receiving daily ω-3 fatty acid infusions. Transl. Anim. Sci 5(4 Suppl 1). doi: 10.1093/tas/txab166

[R13] Bérard J, Kreuzer M, and Bee G (2008). Effect of litter size and birth weight on growth, carcass and pork quality, and their relationship to postmortem proteolysis. J. Anim. Sci 86, 2357–2368. doi: 10.2527/jas.2008-0893 18469061

[R14] Bodell PW, Kodesh E, Haddad F, Zaldivar FP, Cooper DM, and Adams GR (2009). Skeletal muscle growth in young rats is inhibited by chronic exposure to IL-6 but preserved by concurrent voluntary endurance exercise. J. Appl. Physiol. (1985) 106, 443–453. doi: 10.1152/japplphysiol.90831.2008 19057004 PMC2644243

[R15] Boehmer BH, Limesand SW, and Rozance PJ (2017). The impact of IUGR on pancreatic islet development and beta-cell function. J. Endocrinol 235, R63–R76. doi: 10.1530/JOE-17-0076 28808079 PMC5808569

[R16] Boscá L, and Corredor C (1984). Is phosphofructokinase the rate-limiting step of glycolysis? Trends Biochem. Sci 9, 372–373. doi: 10.1016/0968-0004(84)90214-7

[R17] Briana DD, and Malamitsi-Puchner A (2021). Intrauterine growth restriction: the controversial role of perinatal adipocytokines in the prediction of metabolic adult disease. J. Matern. Fetal Neonatal Med 34, 2577–2582. doi: 10.1080/14767058.2019.1669556 31530060

[R18] Brown LD (2014). Endocrine regulation of fetal skeletal muscle growth: impact on future metabolic health. J. Endocrinol 221, R13–R29. doi: 10.1530/JOE-13-0567 24532817 PMC4004098

[R19] Brown LD, Davis M, Wai S, Wesolowski SR, Hay WW Jr., Limesand SW, (2016a). Chronically increased amino acids improve insulin secretion, pancreatic vascularity, and islet size in growth-restricted fetal sheep. Endocrinology 157, 3788–3799. doi: 10.1210/en.2016-1328 27501184 PMC5045508

[R20] Brown LD, Rozance PJ, Bruce JL, Friedman JE, Hay WW Jr., and Wesolowski SR (2015). Limited capacity for glucose oxidation in fetal sheep with intrauterine growth restriction. Am. J. Physiol. Regul. Integr. Comp. Physiol 309, R920–R928. doi: 10.1152/ajpregu.00197.2015 26224688 PMC4666949

[R21] Brown LD, Rozance PJ, Thorn SR, Friedman JE, and Hay WW Jr. (2012). Acute supplementation of amino acids increases net protein accretion in IUGR fetal sheep. Am. J. Physiol. Endocrinol. Metab 303, E352–E364. doi: 10.1152/ajpendo.00059.2012 22649066 PMC3423121

[R22] Brown LD, Wesolowski SR, Kailey J, Bourque S, Wilson A, Andrews SE, (2016b). Chronic hyperinsulinemia increases myoblast proliferation in fetal sheep skeletal muscle. Endocrinology 157, 2447–2460. doi: 10.1210/en.2015-1744 27049667 PMC4891788

[R23] Bruce CR, and Dyck DJ (2004). Cytokine regulation of skeletal muscle fatty acid metabolism: effect of interleukin-6 and tumor necrosis factor-alpha. Am. J. Physiol. Endocrinol. Metab 287, E616–E621. doi: 10.1152/ajpendo.00150.2004 15149950

[R24] Burton GJ, and Jauniaux E (2018). Pathophysiology of placental-derived fetal growth restriction. Am. J. Obstet. Gynecol 218, S745–S761. doi: 10.1016/j.ajog.2017.11.577 29422210

[R25] Cadaret CN (2019). Maternofetal Stress Impairs Skeletal Muscle Growth and Glucose Metabolism in the IUGR-Born Neonatal Lamb; The Effects of Varying the Cognitive Processes Required by Retrieval Practice on the Retention of Knowledge ETD collection for University of Nebraska, Lincoln AAI27543937.

[R26] Cadaret CN, Beede KA, Riley HE, and Yates DT (2017). Acute exposure of primary rat soleus muscle to zilpaterol HCl (β2 adrenergic agonist), TNFα, or IL-6 in culture increases glucose oxidation rates independent of the impact on insulin signaling or glucose uptake. Cytokine 96, 107–113. doi: 10.1016/j.cyto.2017.03.014 28390265 PMC5483180

[R27] Cadaret CN, Merrick EM, Barnes TL, Beede KA, Posont RJ, Petersen JL, (2019). Sustained maternal inflammation during the early third-trimester yields intrauterine growth restriction, impaired skeletal muscle glucose metabolism, and diminished beta-cell function in fetal sheep. J. Anim. Sci 97, 4822–4833. doi: 10.1093/jas/skz321 31616931 PMC6915216

[R28] Cadaret CN, Posont RJ, Beede KA, Riley HE, Loy JD, and Yates DT (2019a). Maternal inflammation at midgestation impairs subsequent fetal myoblast function and skeletal muscle growth in rats, resulting in intrauterine growth restriction at term. Transl. Anim. Sci 3, 867–876. doi: 10.1093/tas/txz037 PMC6476527 31032478

[R29] Cadaret CN, Posont RJ, Swanson RM, Beard JK, Barnes TL, Beede KA, (2019b). Intermittent maternofetal O2 supplementation during late gestation rescues placental insufficiency-induced intrauterine growth restriction and metabolic pathologies in the neonatal lamb. Transl. Anim. Sci 3(Suppl 1), 1696–1700. doi: 10.1093/tas/txz060 33336152 PMC6999172

[R30] Campbell IL, Hobbs MV, Dockter J, Oldstone MB, and Allison J (1994). Islet inflammation and hyperplasia induced by the pancreatic islet-specific overexpression of interleukin-6 in transgenic mice. Am. J. Pathol 145, 157–166. 8030746 PMC1887304

[R31] Cheema IR, Hermann C, Postell S, and Barnes P (2000). Effect of chronic excess of tumour necrosis factor-alpha on contractile proteins in rat skeletal muscle. Cytobios 103, 169–176. 11086712

[R32] Chen X, Kelly AC, Yates DT, Macko AR, Lynch RM, and Limesand SW (2017). Islet adaptations in fetal sheep persist following chronic exposure to high norepinephrine. J. Endocrinol 232, 285–295. doi: 10.1530/JOE-16-0445 27888197 PMC5173394

[R33] Chisaka T, Mogi M, Nakaoka H, Kan-No H, Tsukuda K, Wang XL, (2016). Low-protein diet-induced fetal growth restriction leads to exaggerated proliferative response to vascular injury in postnatal life. Am. J. Hypertens 29, 54–62. doi: 10.1093/ajh/hpv072 26002925

[R34] Contreras-Correa ZE, Messman RD, Sidelinger DR, King EH, Sánchez-Rodríguez HL, Burnett DD, (2021). Melatonin alters bovine uterine artery hemodynamics, vaginal temperatures and fetal morphometrics during late gestational nutrient restriction in a season-dependent manner. J. Anim. Sci 99, skab242. doi: 10.1093/jas/skab242 34387666 PMC8420683

[R35] Cotechini T, and Graham CH (2015). Aberrant maternal inflammation as a cause of pregnancy complications: a potential therapeutic target? Placenta 36, 960–966. doi: 10.1016/j.placenta.2015.05.016 26094029

[R36] Dai C, Huh CG, Thorgeirsson SS, and Liu Y (2005). Beta-cell-specific ablation of the hepatocyte growth factor receptor results in reduced islet size, impaired insulin secretion, and glucose intolerance. Am. J. Pathol 167, 429–436. doi: 10.1016/S0002-9440(10)62987-2 16049329 PMC1603568

[R37] Darendeliler F (2019). IUGR: genetic influences, metabolic problems, environmental associations/triggers, current and future management. Best Pract. Res. Clin. Endocrinol. Metab 33, 101260. doi: 10.1016/j.beem.2019.01.001 30709755

[R38] Davis MA, Camacho LE, Anderson MJ, Steffens NR, Pendleton AL, Kelly AC, (2020). Chronically elevated norepinephrine concentrations lower glucose uptake in fetal sheep. Am. J. Physiol. Regul. Integr. Comp. Physiol 319, R255–R263. doi: 10.1152/ajpregu.00365.2019 32667834 PMC7509250

[R39] Davis MA, Camacho LE, Pendleton AL, Antolic AT, Luna-Ramirez RI, Kelly AC, (2021). Augmented glucose production is not contingent on high catecholamines in fetal sheep with IUGR. J. Endocrinol 249, 195–207. doi: 10.1530/JOE-21-0071 33994373 PMC8175032

[R40] Davis TA, and Fiorotto ML (2009). Regulation of muscle growth in neonates. Curr. Opin. Clin. Nutr. Metab. Care 12, 78–85. doi: 10.1097/MCO.0b013e32831cef9f 19057192 PMC2653196

[R41] Desai M, Gayle DA, Casillas E, Boles J, and Ross MG (2009). Early undernutrition attenuates the inflammatory response in adult rat offspring. J. Matern. Fetal Neonatal Med 22, 571–575. doi: 10.1080/14767050902874105 19488945

[R42] Dinarello CA (2000). Interleukin-18, a proinflammatory cytokine. Eur. Cytokine Netw 11, 483−486. doi: 10.1684/ecn.2006.0047 11203186

[R43] Dwyer CM, Conington J, Corbiere F, Holmoy IH, Muri K, Nowak R, (2016). Invited review: improving neonatal survival in small ruminants: science into practice. Animal 10, 449–459. doi: 10.1017/S1751731115001974 26434788

[R44] Dyck DJ, Heigenhauser GJ, and Bruce CR (2006). The role of adipokines as regulators of skeletal muscle fatty acid metabolism and insulin sensitivity. Acta Physiol. (Oxf) 186, 5–16. doi: 10.1111/j.1748-1716.2005.01502.x 16497175

[R45] Edwards AK, McKnight SM, Askelson K, McKnight JR, Dunlap KA, and Satterfield MC (2020). Adaptive responses to maternal nutrient restriction alter placental transport in ewes. Placenta 96, 1–9. doi: 10.1016/j.placenta.2020.05.002 32421527

[R46] Eifert AW, Wilson ME, Vonnahme KA, Camacho LE, Borowicz PP, Redmer DA, (2015). Effect of melatonin or maternal nutrient restriction on vascularity and cell proliferation in the ovine placenta. Anim. Reprod. Sci 153, 13–21. doi: 10.1016/j.anireprosci.2014.11.022 25578503

[R47] Flinn T, Kleemann DO, Swinbourne AM, Kelly JM, Weaver AC, Walker SK, (2020). Neonatal lamb mortality: major risk factors and the potential ameliorative role of melatonin. J. Anim. Sci. Biotechnol 11, 107. doi: 10.1186/s40104-020-00510-w 33292527 PMC7643391

[R48] Gagnon R (2003). Placental insufficiency and its consequences. Eur. J. Obstet. Gynecol. Reprod. Biol 110(Suppl 1), S99–S107. doi: 10.1016/S0301-2115(03)00179-9 12965097

[R49] Gao F, Ni Y, Luo Z, Liang Y, Yan Z, Xu X, (2012). Atorvastatin attenuates TNF-α-induced increase of glucose oxidation through PGC-1α upregulation in cardiomyocytes. J. Cardiovasc. Pharmacol 59, 500–506. doi: 10.1097/FJC.0b013e31824c853c 22343369

[R50] Gibbs RL, Swanson RM, Beard JK, Schmidt TB, Petersen JL, and Yates DT (2020). Deficits in growth, muscle mass, and body composition following placental insufficiency-induced intrauterine growth restriction persisted in lambs at 60 d of age but were improved by daily clenbuterol supplementation. Transl. Anim. Sci 4(Suppl 1), S53–S57. doi: 10.1093/tas/txaa097 33381721 PMC7754231

[R51] Gibbs RL, Swanson RM, Beard JK, Schmidt TB, Petersen JL, and Yates DT (2021). Deficits in skeletal muscle glucose metabolism and whole-body oxidative metabolism in the IUGR juvenile lamb are improved by daily treatment with clenbuterol. Transl. Anim. Sci 5(4 Suppl 1). doi: 10.1093/tas/txab187

[R52] Greenwood P, Cafe L, Hearnshaw H, and Hennessy D (2005). Consequences of nutrition and growth retardation early in life for growth and composition of cattle and eating quality of beef. Recent Adv. Anim. Nutr. Austr 15, 183–195.

[R53] Greenwood PL, and Bell AW (2003). Consequences of intra-uterine growth retardation for postnatal growth, metabolism and pathophysiology. Reprod. Suppl 61, 195–206. 14635936

[R54] Greenwood PL, and Bell AW (2019). Developmental programming and growth of livestock tissues for meat production. Vet. Clin. North Am. Food Anim. Pract 35, 303–319. doi: 10.1016/j.cvfa.2019.02.008 31103183

[R55] Greenwood PL, and Cafe LM (2007). Prenatal and pre-weaning growth and nutrition of cattle: long-term consequences for beef production. Animal 1, 1283–1296. doi: 10.1017/S175173110700050X 22444884

[R56] Greenwood PL, Hunt AS, Hermanson JW, and Bell AW (2000). Effects of birth weight and postnatal nutrition on neonatal sheep: II. Skeletal muscle growth and development. J. Anim. Sci 78, 50–61. doi: 10.2527/2000.78150x 10682802

[R57] Guo R, Hou W, Dong Y, Yu Z, Stites J, and Weiner CP (2010). Brain injury caused by chronic fetal hypoxemia is mediated by inflammatory cascade activation. Reprod. Sci 17, 540–548. doi: 10.1177/1933719110364061 20360591

[R58] Haddad F, Zaldivar F, Cooper DM, and Adams GR (2005). IL-6-induced skeletal muscle atrophy. J. Appl. Physiol. (1985) 98, 911–917. doi: 10.1152/japplphysiol.01026.2004 15542570

[R59] Hales CN, and Barker DJ (1992). Type 2 (non-insulin-dependent) diabetes mellitus: the thrifty phenotype hypothesis. Diabetologia 35, 595–601. doi: 10.1007/BF00400248 1644236

[R60] Hales CN, Barker DJ, Clark PM, Cox LJ, Fall C, Osmond C, (1991). Fetal and infant growth and impaired glucose tolerance at age 64. BMJ 303, 1019–1022. doi: 10.1136/bmj.303.6809.1019 1954451 PMC1671766

[R61] Harms RZ, Yarde DN, Guinn Z, Lorenzo-Arteaga KM, Corley KP, Cabrera MS, (2015). Increased expression of IL-18 in the serum and islets of type 1 diabetics. Mol. Immunol 64, 306–312. doi: 10.1016/j.molimm.2014.12.012 25576800 PMC4315765

[R62] Hudalla H, Karenberg K, Kuon RJ, Pöschl J, Tschada R, and Frommhold D (2018). LPS-induced maternal inflammation promotes fetal leukocyte recruitment and prenatal organ infiltration in mice. Pediatr. Res 84, 757–764. doi: 10.1038/s41390-018-0030-z 30135596

[R63] Jensen CB, Storgaard H, Dela F, Holst JJ, Madsbad S, and Vaag AA (2002). Early differential defects of insulin secretion and action in 19-year-old caucasian men who had low birth weight. Diabetes 51, 1271–1280. doi: 10.2337/diabetes.51.4.1271 11916955

[R64] Ji Y, Wu Z, Dai Z, Wang X, Li J, Wang B, (2017). Fetal and neonatal programming of postnatal growth and feed efficiency in swine. J. Anim. Sci. Biotechnol 8, 42. doi: 10.1186/s40104-017-0173-5 28484595 PMC5420136

[R65] Johansson A, Lau J, Sandberg M, Borg LA, Magnusson PU, and Carlsson PO (2009). Endothelial cell signalling supports pancreatic beta cell function in the rat. Diabetologia 52, 2385–2394. doi: 10.1007/s00125-009-1485-6 19669728

[R66] Johnson DE, O’Keefe RA, and Grandis JR (2018). Targeting the IL-6/JAK/STAT3 signalling axis in cancer. Nat. Rev. Clin. Oncol 15, 234–248. doi: 10.1038/nrclinonc.2018.8 29405201 PMC5858971

[R67] Kelly AC, Bidwell CA, McCarthy FM, Taska DJ, Anderson MJ, Camacho LE, (2017). RNA sequencing exposes adaptive and immune responses to intrauterine growth restriction in fetal sheep islets. Endocrinology 158, 743–755. doi: 10.1210/en.2016-1901 28200173 PMC5460795

[R68] Kemp MW (2014). Preterm birth, intrauterine infection, and fetal inflammation. Front. Immunol 5, 574. doi: 10.3389/fimmu.2014.00574 25520716 PMC4249583

[R69] Kesavan K, and Devaskar SU (2019). Intrauterine growth restriction: postnatal monitoring and outcomes. Pediatr. Clin. North Am 66, 403–423. doi: 10.1016/j.pcl.2018.12.009 30819345

[R70] Kirchofer KS, Calkins CB, and Gwartney BL (2002). Fiber-type composition of muscles of the beef chuck and round. J. Anim. Sci 80, 2872–2878. doi: 10.2527/2002.80112872x 12462254

[R71] Knudsen JG, Gudiksen A, Bertholdt L, Overby P, Villesen I, Schwartz CL, (2017). Skeletal muscle IL-6 regulates muscle substrate utilization and adipose tissue metabolism during recovery from an acute bout of exercise. PLoS ONE 12, e0189301. doi: 10.1371/journal.pone.0189301 29253016 PMC5734691

[R72] Krajewski P, Sieroszewski P, Karowicz-Bilinska A, Kmiecik M, Chudzik A, Strzalko-Gloskowska B, (2014). Assessment of interleukin-6, interleukin-8 and interleukin-18 count in the serum of IUGR newborns. J. Matern. Fetal Neonatal Med 27, 1142–1145. doi: 10.3109/14767058.2013.851186 24093539

[R73] Kubota T, Kubota N, Kumagai H, Yamaguchi S, Kozono H, Takahashi T, (2011). Impaired insulin signaling in endothelial cells reduces insulin-induced glucose uptake by skeletal muscle. Cell Metab 13, 294–307. doi: 10.1016/j.cmet.2011.01.018 21356519

[R74] Lacey TA, Gibbs RL, Most MS, Beer HN, Hicks ZH, Grijalva PC, Petersen JL, and Yates DT (2021). Decreased fetal biometrics and impaired β cell function in IUGR fetal sheep are improved by daily ω-3 PUFA infusion. Transl. Anim. Sci 5(4 Suppl 1). doi: 10.1093/tas/txab168

[R75] Lackman F, Capewell V, Gagnon R, and Richardson B (2001). Fetal umbilical cord oxygen values and birth to placental weight ratio in relation to size at birth. Am. J. Obstet. Gynecol 185, 674–682. doi: 10.1067/mob.2001.116686 11568797

[R76] Lang CH, Frost RA, Nairn AC, MacLean DA, and Vary TC (2002). TNF-alpha impairs heart and skeletal muscle protein synthesis by altering translation initiation. Am. J. Physiol. Endocrinol. Metab 282, E336–E347. doi: 10.1152/ajpendo.00366.2001 11788365

[R77] Lemley CO, Meyer AM, Camacho LE, Neville TL, Newman DJ, Caton JS, (2012). Melatonin supplementation alters uteroplacental hemodynamics and fetal development in an ovine model of intrauterine growth restriction. Am. J. Physiol. Regul. Integr. Comp. Physiol 302, R454–R467. doi: 10.1152/ajpregu.00407.2011 22129617

[R78] Li W, Moylan JS, Chambers MA, Smith J, and Reid MB (2009). Interleukin-1 stimulates catabolism in C2C12 myotubes. Am. J. Physiol. Cell Physiol 297, C706–C714. doi: 10.1152/ajpcell.00626.2008 19625606 PMC2740393

[R79] Li YP, and Reid MB (2001). Effect of tumor necrosis factor-alpha on skeletal muscle metabolism. Curr. Opin. Rheumatol 13, 483–487. doi: 10.1097/00002281-200111000-00005 11698724

[R80] Limesand SW, Jensen J, Hutton J, and Hay WW Jr. (2005). Diminished beta-cell replication contributes to reduced beta-cell mass in fetal sheep with intrauterine growth restriction. Am. J. Physiol. Regul. Integr. Comp. Physiol 288, R1297–R305. doi: 10.1152/ajpregu.00494.2004 15650129

[R81] Limesand SW, and Rozance PJ (2017). Fetal adaptations in insulin secretion result from high catecholamines during placental insufficiency. J. Physiol 595, 5103–5113. doi: 10.1113/JP273324 28194805 PMC5538202

[R82] Limesand SW, Rozance PJ, Smith D, and Hay WW Jr. (2007). Increased insulin sensitivity and maintenance of glucose utilization rates in fetal sheep with placental insufficiency and intrauterine growth restriction. Am. J. Physiol. Endocrinol. Metab 293, E1716–E1725. doi: 10.1152/ajpendo.00459.2007 17895285

[R83] Limesand SW, Rozance PJ, Zerbe GO, Hutton JC, and Hay WW Jr. (2006). Attenuated insulin release and storage in fetal sheep pancreatic islets with intrauterine growth restriction. Endocrinology 147, 1488–1497. doi: 10.1210/en.2005-0900 16339204

[R84] Liu J, and He J (2014). Effects of birth weight and postnatal high-fat diet on growth performance, carcass and meat quality in pigs. J. Anim. Plant Sci 24, 1606–1612.

[R85] Lo J, Bernstein LE, Canavan B, Torriani M, Jackson MB, Ahima RS, (2007). Effects of TNF-alpha neutralization on adipocytokines and skeletal muscle adiposity in the metabolic syndrome. Am. J. Physiol. Endocrinol. Metab 293, E102–E109. doi: 10.1152/ajpendo.00089.2007 17374698 PMC3196534

[R86] Macko AR, Yates DT, Chen X, Shelton LA, Kelly AC, Davis MA, (2016). Adrenal demedullation and oxygen supplementation independently increase glucose-stimulated insulin concentrations in fetal sheep with intrauterine growth restriction. Endocrinology 157, 2104–2115. doi: 10.1210/en.2015-1850 26937714 PMC4870878

[R87] Maier A, McEwan JC, Dodds KG, Fischman DA, Fitzsimons RB, and Harris AJ (1992). Myosin heavy chain composition of single fibres and their origins and distribution in developing fascicles of sheep tibialis cranialis muscles. J. Musc. Res. Cell Mobil 13, 551–572. doi: 10.1007/BF01737997 1460083

[R88] Makikallio K, Kaukola T, Tuimala J, S. FK, Hallman M, and Ojaniemi M (2012). Umbilical artery chemokine CCL16 is associated with preterm preeclampsia and fetal growth restriction. Cytokine 60, 377–384. doi: 10.1016/j.cyto.2012.07.008 22857868

[R89] Matyba P, Florowski T, Dasiewicz K, Ferenc K, Olszewski J, Trela M, (2021). Performance and meat quality of intrauterine growth restricted pigs. Animals 11, 254. doi: 10.3390/ani11020254 33498468 PMC7909567

[R90] Mericq V, Ong KK, Bazaes R, Pena V, Avila A, Salazar T, (2005). Longitudinal changes in insulin sensitivity and secretion from birth to age three years in small- and appropriate-for-gestational-age children. Diabetologia 48, 2609–2614. doi: 10.1007/s00125-005-0036-z 16283238

[R91] Morrison JL (2008). Sheep models of intrauterine growth restriction: fetal adaptations and consequences. Clin. Exp. Pharmacol. Physiol 35, 730–743. doi: 10.1111/j.1440-1681.2008.04975.x 18498533

[R92] Nardozza LM, Caetano AC, Zamarian AC, Mazzola JB, Silva CP, Marcal VM, (2017). Fetal growth restriction: current knowledge. Arch. Gynecol. Obstet 295, 1061–1077. doi: 10.1007/s00404-017-4341-9 28285426

[R93] Nawabi MD, Block KP, Chakrabarti MC, and Buse MG (1990). Administration of endotoxin, tumor necrosis factor, or interleukin 1 to rats activates skeletal muscle branched-chain alpha-keto acid dehydrogenase. J. Clin. Invest 85, 256–263. doi: 10.1172/JCI114421 2404025 PMC296413

[R94] Neel JV (1962). Diabetes mellitus: a “thrifty” genotype rendered detrimental by “progress”? Am. J. Hum. Genet 14, 353–362. 13937884 PMC1932342

[R95] Niu Y, He J, Ahmad H, Shen M, Zhao Y, Gan Z, (2019a). Dietary curcumin supplementation increases antioxidant capacity, upregulates Nrf2 and Hmox1 levels in the liver of piglet model with intrauterine growth retardation. Nutrients 11, 2978. doi: 10.3390/nu11122978 PMC6950043 31817533

[R96] Niu Y, He J, Ahmad H, Wang C, Zhong X, Zhang L, (2019b). Curcumin attenuates insulin resistance and hepatic lipid accumulation in a rat model of intra-uterine growth restriction through insulin signalling pathway and sterol regulatory element binding proteins. Br. J. Nutr 122, 616–624. doi: 10.1017/S0007114519001508 31237229

[R97] Niu Y, He J, Zhao Y, Shen M, Zhang L, Zhong X, (2019c). Effect of curcumin on growth performance, inflammation, insulin level, and lipid metabolism in weaned piglets with IUGR. Animals (Basel) 9, 1098. doi: 10.3390/ani9121098 PMC6940831 31818040

[R98] Oh JW, Park CW, Moon KC, Park JS, and Jun JK (2019). The relationship among the progression of inflammation in umbilical cord, fetal inflammatory response, early-onset neonatal sepsis, and chorioamnionitis. PLoS ONE 14, e0225328. doi: 10.1371/journal.pone.0225328 31743377 PMC6863554

[R99] Oleson B, McGraw J, Broniowska K, Annamalai M, Chen J, Bushkofsky J, (2015). Distinct differences in the responses of the human pancreatic β-cell line EndoC-βH1 and human islets to proinflammatory cytokines. Amer. J. Physiol. Regul. Integr. Comp. Physiol 309, R525–R534. doi: 10.1152/ajpregu.00544.2014 26084699 PMC4591379

[R100] Oliveira V, Silva Junior SD, de Carvalho MH, Akamine EH, Michelini LC, and Franco MC (2017). Intrauterine growth restriction increases circulating mitochondrial DNA and Toll-like receptor 9 expression in adult offspring: could aerobic training counteract these adaptations? J. Dev. Orig. Health Dis 8, 236–243. doi: 10.1017/S2040174416000714 28004624

[R101] Ong KK, Petry CJ, Emmett PM, Sandhu MS, Kiess W, Hales CN, (2004). Insulin sensitivity and secretion in normal children related to size at birth, postnatal growth, and plasma insulin-like growth factor-I levels. Diabetologia 47, 1064–1070. doi: 10.1007/s00125-004-1405-8 15156313

[R102] Otis JS, Niccoli S, Hawdon N, Sarvas JL, Frye MA, Chicco AJ, (2014). Pro-inflammatory mediation of myoblast proliferation. PLoS ONE 9, e92363. doi: 10.1371/journal.pone.0092363 24647690 PMC3960233

[R103] Pantham P, Rosario FJ, Weintraub ST, Nathanielsz PW, Powell TL, Li C, (2016). Down-regulation of placental transport of amino acids precedes the development of intrauterine growth restriction in maternal nutrient restricted baboons. Biol. Reprod 95, 98. doi: 10.1095/biolreprod.116.141085 27605346 PMC5178152

[R104] Pendleton AL, Antolic AT, Kelly AC, Davis MA, Camacho LE, Doubleday K, (2020). Lower oxygen consumption and complex I activity in mitochondria isolated from skeletal muscle of fetal sheep with intrauterine growth restriction. Am. J. Physiol. Endocrinol. Metab 319, E67–E80. doi: 10.1152/ajpendo.00057.2020 32396498 PMC7468780

[R105] Pendleton AL, Humphreys LR, Davis MA, Camacho LE, Anderson MJ, and Limesand SW (2019). Increased pyruvate dehydrogenase activity in skeletal muscle of growth-restricted ovine fetuses. Am. J. Physiol. Regul. Integr. Comp. Physiol 317, R513–r20. doi: 10.1152/ajpregu.00106.2019 31314546 PMC6842904

[R106] Pendleton AL, Wesolowski SR, Regnault TRH, Lynch RM, and Limesand SW (2021). Dimming the powerhouse: mitochondrial dysfunction in the liver and skeletal muscle of intrauterine growth restricted fetuses. Front. Endocrinol 12, 612888. doi: 10.3389/fendo.2021.612888 PMC8165279 34079518

[R107] Plomgaard P, Penkowa M, and Pedersen BK (2005). Fiber type specific expression of TNF-alpha, IL-6 and IL-18 in human skeletal muscles. Exerc. Immunol. Rev 11, 53–63. 16385844

[R108] Popa C, Netea MG, van Riel PL, van der Meer JW, and Stalenhoef AF (2007). The role of TNF-alpha in chronic inflammatory conditions, intermediary metabolism, and cardiovascular risk. J. Lipid Res 48, 751–762. doi: 10.1194/jlr.R600021-JLR200 17202130

[R109] Posont R, Beede K, Limesand S, and Yates D (2018). Changes in myoblast responsiveness to TNFa and IL-6 contribute to decreased skeletal muscle mass in intrauterine growth restricted fetal sheep. Transl. Anim. Sci 2(Suppl 1), S44–S47. doi: 10.1093/tas/txy038 30627704 PMC6310364

[R110] Posont RJ (2019). The Role of Inflammatory Pathways in Development, Growth, and Metabolism of Skeletal Muscle in IUGR Offspring; Blood Gene Expression of Inflammatory Factors as Novel Biomarkers for Assessing Stress and Wellbeing in Exotic Species MS Thesis, Department of Animal Science, University of Nebraska-Lincoln.

[R111] Posont RJ, Cadaret CN, Barnes TL, and Yates DT (2017). A potential role for mTORC1/2 in β2 adrenergic regulation of skeletal muscle glucose oxidation in models of intrauterine growth restriction. Diabesity 3, 9–12. doi: 10.15562/diabesity.2017.40 33834090 PMC8025757

[R112] Posont RJ, Cadaret CN, Beard JK, Swanson RM, Gibbs RL, Marks-Nelson ES, (2021). Maternofetal inflammation induced for 2 wk in late gestation reduced birth weight and impaired neonatal growth and skeletal muscle glucose metabolism in lambs. J. Anim. Sci 99, skab102. doi: 10.1093/jas/skab102 33780540 PMC8269969

[R113] Posont RJ, and Yates DT (2019). Postnatal nutrient repartitioning due to adaptive developmental programming. Vet. Clin. North Am. Food Anim. Pract 35, 277–288. doi: 10.1016/j.cvfa.2019.02.001 31103181 PMC6527338

[R114] Poudel R, McMillen IC, Dunn SL, Zhang S, and Morrison JL (2015). Impact of chronic hypoxemia on blood flow to the brain, heart, and adrenal gland in the late-gestation IUGR sheep fetus. Am. J. Physiol. Regul. Integr. Comp. Physiol 308, R151–R162. doi: 10.1152/ajpregu.00036.2014 25427766

[R115] Raychaudhuri N, Raychaudhuri S, Thamotharan M, and Devaskar SU (2008). Histone code modifications repress glucose transporter 4 expression in the intrauterine growth-restricted offspring. J. Biol. Chem 283, 13611–13626. doi: 10.1074/jbc.M800128200 18326493 PMC2376250

[R116] Regnault TR, de Vrijer B, Galan HL, Davidsen ML, Trembler KA, Battaglia FC, (2003). The relationship between transplacental O_2_ diffusion and placental expression of PlGF, VEGF and their receptors in a placental insufficiency model of fetal growth restriction. J. Physiol 550, 641–656. doi: 10.1113/jphysiol.2003.039511 12740423 PMC2343042

[R117] Regnault TR, Friedman JE, Wilkening RB, Anthony RV, and Hay WW Jr. (2005). Fetoplacental transport and utilization of amino acids in IUGR–a review. Placenta 26(Suppl A), S52–S62. doi: 10.1016/j.placenta.2005.01.003 15837069

[R118] Reid MB, and Li YP (2001). Cytokines and oxidative signalling in skeletal muscle. Acta Physiol. Scand 171, 225–232. doi: 10.1046/j.1365-201x.2001.00824.x 11412134

[R119] Remels AH, Gosker HR, Verhees KJ, Langen RC, and Schols AM (2015). TNF-alpha-induced NF-kappaB activation stimulates skeletal muscle glycolytic metabolism through activation of HIF-1alpha. Endocrinology 156, 1770–1781. doi: 10.1210/en.2014-1591 25710281

[R120] Reynolds LP, Borowicz PP, Caton JS, Crouse MS, Dahlen CR, and Ward AK (2019). Developmental programming of fetal growth and development. Vet. Clin. North Am. Food Anim. Pract 35, 229–247. doi: 10.1016/j.cvfa.2019.02.006 31103178

[R121] Reynolds LP, Borowicz PP, Caton JS, Vonnahme KA, Luther JS, Hammer CJ, (2010). Developmental programming: the concept, large animal models, and the key role of uteroplacental vascular development. J. Anim. Sci 88(13 Suppl), E61–E72. doi: 10.2527/jas.2009-2359 20023136

[R122] Rhoades RD, King DA, Jenschke BE, Behrends JM, Hively TS, and Smith SB (2005). Postmortem regulation of glycolysis by 6-phosphofructokinase in bovine *M. Sternocephalicus pars mandibularis*. Meat. Sci 70, 621–626. doi: 10.1016/j.meatsci.2005.01.024 22063888

[R123] Riddle ES, Campbell MS, Lang BY, Bierer R, Wang Y, Bagley HN, (2014). Intrauterine growth restriction increases TNF alpha and activates the unfolded protein response in male rat pups. J. Obes 2014, 829862. doi: 10.1155/2014/829862 24804087 PMC3997936

[R124] Roberge S, Nicolaides K, Demers S, Hyett J, Chaillet N, and Bujold E (2017). The role of aspirin dose on the prevention of preeclampsia and fetal growth restriction: systematic review and meta-analysis. Am. J. Obstet. Gynecol 216, 110.e6–120.e6. doi: 10.1016/j.ajog.2016.09.076 27640943

[R125] Robinson DL, Cafe LM, and Greenwood PL (2013). Meat Science and Muscle Biology Symposium: developmental programming in cattle: consequences for growth, efficiency, carcass, muscle, and beef quality characteristics. J. Anim. Sci 91, 1428–1442. doi: 10.2527/jas.2012-5799 23230118

[R126] Romero R, Gotsch F, Pineles B, and Kusanovic JP (2007). Inflammation in pregnancy: its roles in reproductive physiology, obstetrical complications, and fetal injury. Nutr. Rev 65(12 Pt 2), S194–S202. doi: 10.1301/nr.2007.dec.S194-S202 18240548

[R127] Rozance PJ, Anderson M, Martinez M, Fahy A, Macko AR, Kailey J, (2015). Placental insufficiency decreases pancreatic vascularity and disrupts hepatocyte growth factor signaling in the pancreatic islet endothelial cell in fetal sheep. Diabetes 64, 555–564. doi: 10.2337/db14-0462 25249573 PMC4303968

[R128] Rozance PJ, Zastoupil L, Wesolowski SR, Goldstrohm DA, Strahan B, Cree-Green M, (2018). Skeletal muscle protein accretion rates and hindlimb growth are reduced in late gestation intrauterine growth-restricted fetal sheep. J. Physiol 596, 67–82. doi: 10.1113/JP275230 28940557 PMC5746524

[R129] Saghizadeh M, Ong JM, Garvey WT, Henry RR, and Kern PA (1996). The expression of TNF alpha by human muscle. Relationship to insulin resistance. J. Clin. Invest 97, 1111–1116. doi: 10.1172/JCI118504 8613535 PMC507159

[R130] Saleem T, Sajjad N, Fatima S, Habib N, Ali S, and Qadir M (2011). Intrauterine growth retardation - small events, big consequences. Ital. J. Pediatr 37, 41. doi: 10.1186/1824-7288-37-41 21899747 PMC3177763

[R131] Sedgh G, Singh S, and Hussain R (2014). Intended and unintended pregnancies worldwide in 2012 and recent trends. Stud. Fam. Plann 45, 301–314. doi: 10.1111/j.1728-4465.2014.00393.x 25207494 PMC4727534

[R132] Sharma D, Farahbakhsh N, Shastri S, and Sharma P (2016a). Intrauterine growth restriction - part 2. J. Matern. Fetal Neonatal Med 29, 4037–4048. doi: 10.3109/14767058.2016.1154525 26979578

[R133] Sharma D, Sharma P, and Shastri S (2017). Genetic, metabolic and endocrine aspect of intrauterine growth restriction: an update. J. Matern. Fetal Neonatal Med 30, 2263–2275. doi: 10.1080/14767058.2016.1245285 27718783

[R134] Sharma D, Shastri S, Farahbakhsh N, and Sharma P (2016b). Intrauterine growth restriction - part 1. J. Matern. Fetal Neonatal Med 29, 3977–3987. doi: 10.3109/14767058.2016.1152249 26856409

[R135] Soto N, Bazaes RA, Pena V, Salazar T, Avila A, Iniguez G, (2003). Insulin sensitivity and secretion are related to catch-up growth in small-forgestational-age infants at age 1 year: results from a prospective cohort. J. Clin. Endocrinol. Metab 88, 3645–3650. doi: 10.1210/jc.2002-030031 12915649

[R136] Soto SM, Blake AC, Wesolowski SR, Rozance PJ, Barthel KB, Gao B, (2017). Myoblast replication is reduced in the IUGR fetus despite maintained proliferative capacity *in vitro*. J. Endocrinol 232, 475–491. doi: 10.1530/JOE-16-0123 28053000 PMC5440081

[R137] Stremming J, Jansson T, Powell TL, Rozance PJ, and Brown LD (2020). Reduced Na(+) K(+) -ATPase activity may reduce amino acid uptake in intrauterine growth restricted fetal sheep muscle despite unchanged *ex vivo* amino acid transporter activity. J. Physiol 598, 1625–1639. doi: 10.1113/JP278933 31909825 PMC9004792

[R138] Sumitani S, Goya K, Testa JR, Kouhara H, and Kasayama S (2002). Akt1 and Akt2 Differently regulate muscle creatine kinase and myogenin gene transcription in insulin-induced differentiation of C2C12 myoblasts. Endocrinology 143, 820–828. doi: 10.1210/endo.143.3.8687 11861503

[R139] Sutton GM, Centanni AV, and Butler AA (2010). Protein malnutrition during pregnancy in C57BL/6J mice results in offspring with altered circadian physiology before obesity. Endocrinology 151, 1570–1580. doi: 10.1210/en.2009-1133 20160133 PMC2850243

[R140] Swanson RM, Tait RG, Galles BM, Duffy EM, Schmidt TB, Petersen JL, (2020). Heat stress-induced deficits in growth, metabolic efficiency, and cardiovascular function coincided with chronic systemic inflammation and hypercatecholaminemia in ractopamine-supplemented feedlot lambs. J. Anim. Sci 98, skaa168. doi: 10.1093/jas/skaa168 32428228 PMC7320631

[R141] Tarry-Adkins JL, Fernandez-Twinn DS, Hargreaves IP, Neergheen V, Aiken CE, Martin-Gronert MS, (2016). Coenzyme Q10 prevents hepatic fibrosis, inflammation, and oxidative stress in a male rat model of poor maternal nutrition and accelerated postnatal growth. Am. J. Clin. Nutr 103, 579–588. doi: 10.3945/ajcn.115.119834 26718412 PMC4733260

[R142] Thorn SR, Regnault TR, Brown LD, Rozance PJ, Keng J, Roper M, (2009). Intrauterine growth restriction increases fetal hepatic gluconeogenic capacity and reduces messenger ribonucleic acid translation initiation and nutrient sensing in fetal liver and skeletal muscle. Endocrinology 150, 3021–3030. doi: 10.1210/en.2008-1789 19342452 PMC2703533

[R143] Tracey KJ, and Cerami A (1993). Tumor necrosis factor, other cytokines and disease. Annu. Rev. Cell Biol 9, 317–343. doi: 10.1146/annurev.cb.09.110193.001533 8280464

[R144] Visentin S, Lapolla A, Londero AP, Cosma C, Dalfra M, Camerin M, (2014). Adiponectin levels are reduced while markers of systemic inflammation and aortic remodelling are increased in intrauterine growth restricted mother-child couple. Biomed Res. Int 2014, 401595. doi: 10.1155/2014/401595 25045669 PMC4090565

[R145] Wai SG, Rozance PJ, Wesolowski SR, Hay WW Jr., and Brown LD (2018). Prolonged amino acid infusion into intrauterine growth restricted fetal sheep increases leucine oxidation rates. Am. J. Physiol. Endocrinol. Metab 315, E1143–E1153. doi: 10.1152/ajpendo.00128.2018 30205012 PMC6336957

[R146] Wang J, Cao M, Yang M, Lin Y, Che L, Fang Z, (2016). Intra-uterine undernutrition amplifies age-associated glucose intolerance in pigs via altered DNA methylation at muscle GLUT4 promoter. Br. J. Nutr 116, 390–401. doi: 10.1017/S0007114516002166 27265204

[R147] Wang J, Platt A, Upmanyu R, Germer S, Lei G, Rabe C, (2013). IL-6 pathway-driven investigation of response to IL-6 receptor inhibition in rheumatoid arthritis. BMJ Open 3, e003199. doi: 10.1136/bmjopen-2013-003199 PMC3753518 23959753

[R148] Wilson SJ, McEwan JC, Sheard PW, and Harris AJ (1992). Early stages of myogenesis in a large mammal: formation of successive generations of myotubes in sheep tibialis cranialis muscle. J. Musc. Res. Cell Mobil 13, 534–550. doi: 10.1007/BF01737996 1460082

[R149] Wolsk E, Mygind H, Grøndahl TS, Pedersen BK, and van Hall G (2010). IL-6 selectively stimulates fat metabolism in human skeletal muscle. Am. J. Physiol. Endocrinol. Metab 299, E832–E840. doi: 10.1152/ajpendo.00328.2010 20823453

[R150] Wu G, Bazer F, Wallace J, and Spencer T (2006). Board-invited review: intrauterine growth retardation: implications for the animal sciences. J. Anim. Sci 84, 2316–2337. doi: 10.2527/jas.2006-156 16908634

[R151] Xing Y, Wei H, Xiao X, Chen Z, Liu H, Tong X, (2020). Methylated Vnn1 at promoter regions induces asthma occurrence via the PI3K/Akt/NFκB-mediated inflammation in IUGR mice. Biol. Open 9, bio049106. doi: 10.1242/bio.049106 32139393 PMC7197710

[R152] Yates DT, Cadaret CN, Beede KA, Riley HE, Macko AR, Anderson MJ, (2016). Intrauterine growth-restricted sheep fetuses exhibit smaller hindlimb muscle fibers and lower proportions of insulin-sensitive Type I fibers near term. Am. J. Physiol. Regul. Integr. Comp. Physiol 310, R1020–R1029. doi: 10.1152/ajpregu.00528.2015 27053651 PMC4935506

[R153] Yates DT, Camacho LE, Kelly AC, Steyn LV, Davis MA, Antolic AT, (2019). Postnatal beta2 adrenergic treatment improves insulin sensitivity in lambs with IUGR but not persistent defects in pancreatic islets or skeletal muscle. J. Physiol 597, 5835–5858. doi: 10.1113/JP278726 31665811 PMC6911010

[R154] Yates DT, Clarke DS, Macko AR, Anderson MJ, Shelton LA, Nearing M, (2014). Myoblasts from intrauterine growth-restricted sheep fetuses exhibit intrinsic deficiencies in proliferation that contribute to smaller semitendinosus myofibres. J. Physiol 592, 3113–3125. doi: 10.1113/jphysiol.2014.272591 24860171 PMC4214663

[R155] Yates DT, Macko AR, Nearing M, Chen X, Rhoads RP, and Limesand SW (2012). Developmental programming in response to intrauterine growth restriction impairs myoblast function and skeletal muscle metabolism. J. Pregnancy 2012, 631038. doi: 10.1155/2012/631038 22900186 PMC3415084

[R156] Yates DT, Petersen JL, Schmidt TB, Cadaret CN, Barnes TL, Posont RJ, (2018). ASAS-SSR triennnial reproduction symposium: looking back and moving forward-how reproductive physiology has evolved: fetal origins of impaired muscle growth and metabolic dysfunction: lessons from the heat-stressed pregnant ewe. J. Anim. Sci 96, 2987–3002. doi: 10.1093/jas/sky164 29701769 PMC6095381

[R157] Yin H, Price F, and Rudnicki MA (2013). Satellite cells and the muscle stem cell niche. Physiol. Rev 93, 23–67. doi: 10.1152/physrev.00043.2011 23303905 PMC4073943

[R158] Youd JM, Rattigan S, and Clark MG (2000). Acute impairment of insulin-mediated capillary recruitment and glucose uptake in rat skeletal muscle *in vivo* by TNF-alpha. Diabetes 49, 1904–1909. doi: 10.2337/diabetes.49.11.1904 11078458

[R159] Zhao M, Chen YH, Dong XT, Zhou J, Chen X, Wang H, (2013). Folic acid protects against lipopolysaccharide-induced preterm delivery and intrauterine growth restriction through its anti-inflammatory effect in mice. PLoS ONE 8, e82713. doi: 10.1371/journal.pone.0082713 24324824 PMC3855776

